# Supplementary figures and images for: Thyroid hormone deficiency during zebrafish development impairs central nervous system myelination
Source: PLoS One. 2021 Aug 17;16(8):e0256207. doi: 10.1371/journal.pone.0256207 (PMC8370640; doi:10.1371/journal.pone.0256207)

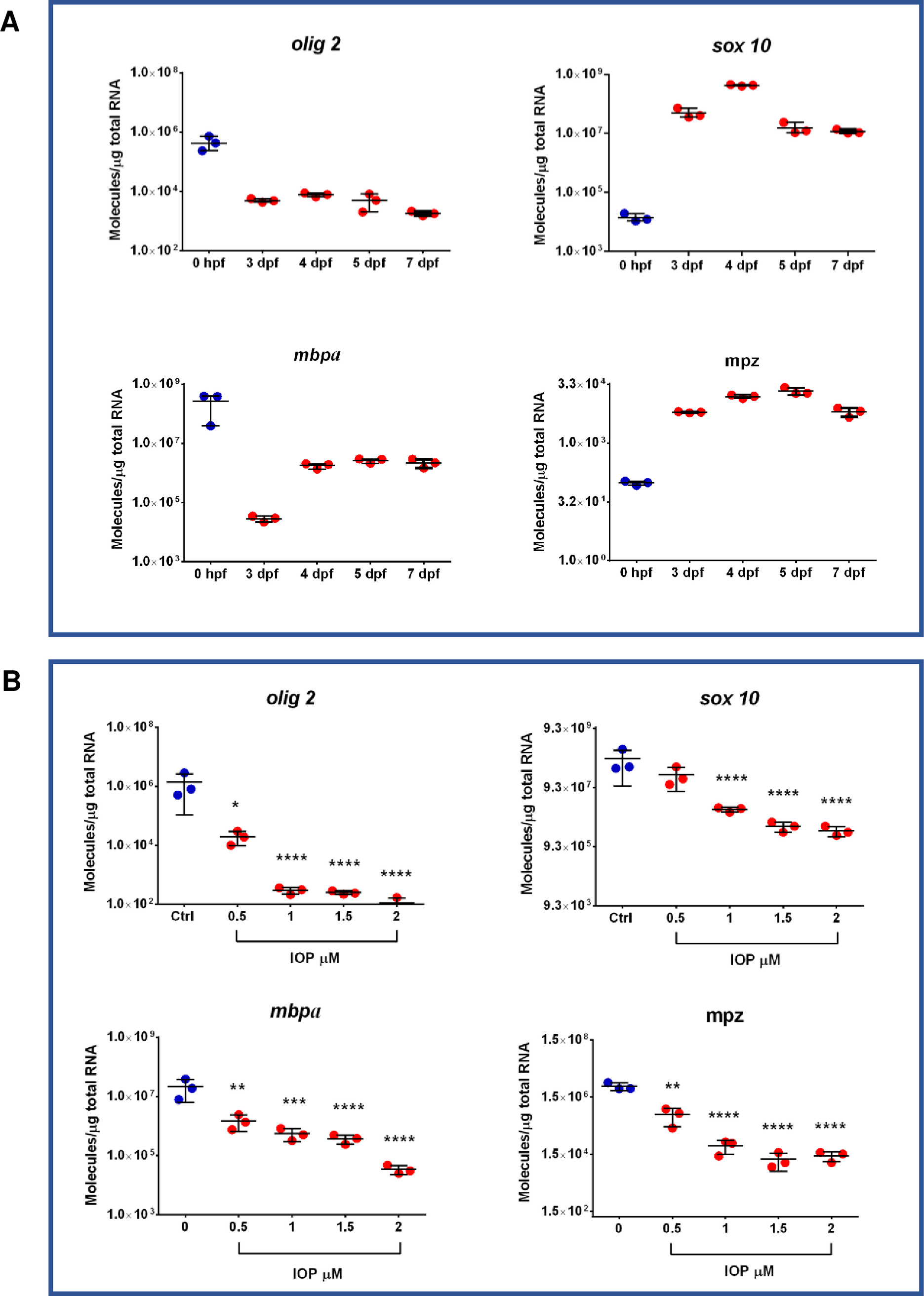

Supplement: S1 Fig — A. mRNA quantification at 0 hpf, 3dpf, 4dpf, 5dpf and 7dpf. Results are represented on a logarithmic scale (mean ± SEM; n = 3 pools of 50–60 larvae per stage). B. 7 dpf zebrafish larvae were exposed to 0.5 μM, 1 μM, 1.5 μM and 2 μM IOP and mRNA expression of olig2, sox10, mbpa and mpz genes was quantified. Statistical analysis was performed with one-way ANOVA coupled with Tukey’s multiple comparison test with respect to the control groups. Significant differences are indicated as *p <0.05, **p <0.01, ***p <0.001 and ****p<0.0001. (TIF) [file pone.0256207.s001.tif]

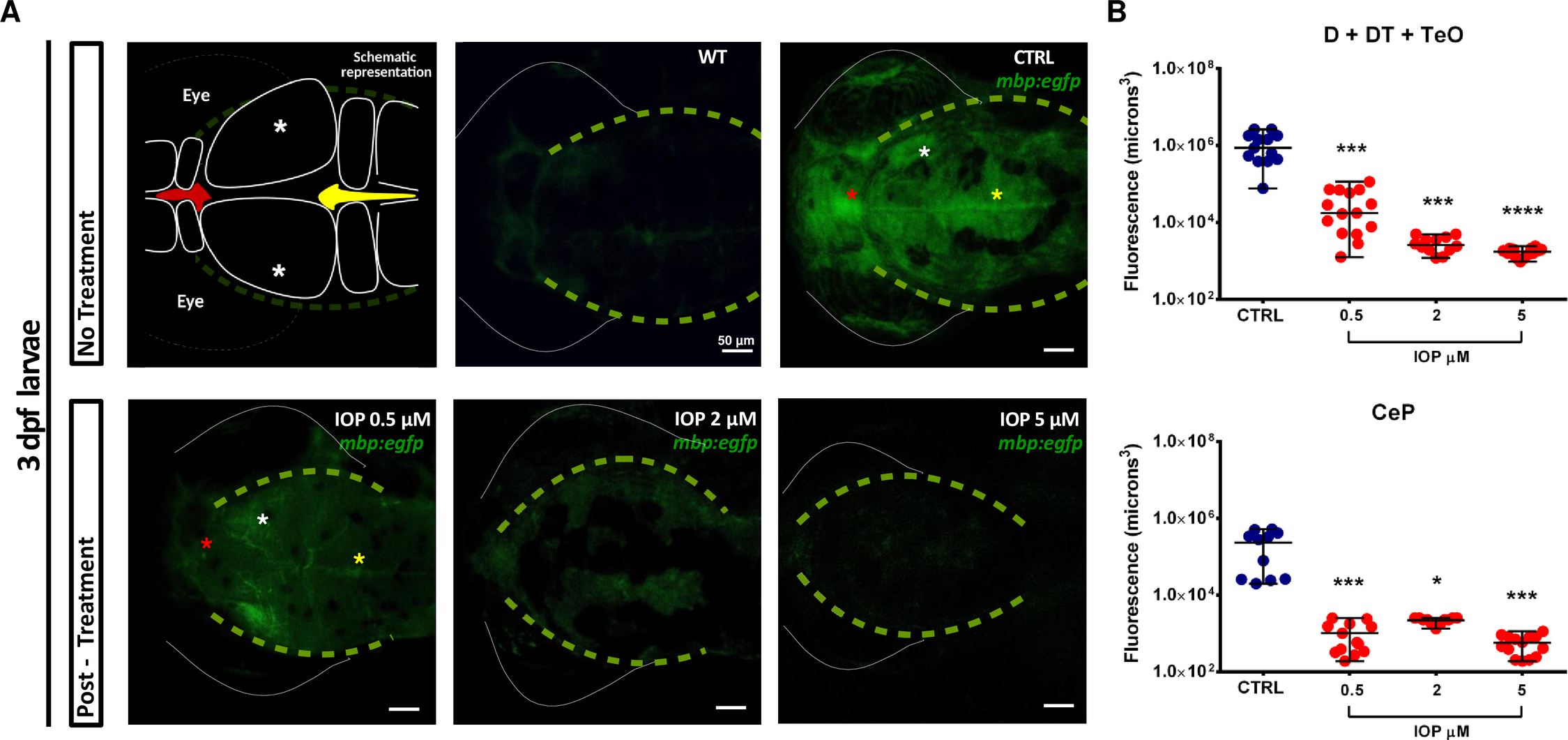

Supplement: S2 Fig — (A) Schematic drawing with dorsal view of the head. The dashed lines mark the brain of the larva (green) and the eyes (white). Red and yellow asterisks refer to the regions that make the division between Tel: Telencephalon, TeO: Tectum opticum, CeP: Cerebellar plate and MO: Medulla oblongata. Confocal images of the larval brain without treatment and with IOP 0.5, 2 and 5 μM treatment are shown. The images are the representation of the maximum intensity projection of the entire set of Z-stacks. (B) The graphs show the volume of the myelinated area in voxels. Data are shown as individual values and mean ± SEM (approximately n = 13 larvae/group). Statistical analysis between medians was performed using one-way ANOVA coupled with Tukey’s multiple comparison test. Significant differences (CTRL vs Treatment) are indicated as *p< 0.05, ***p< 0.001 and ****p< 0.0001. (TIF) [file pone.0256207.s002.tif]

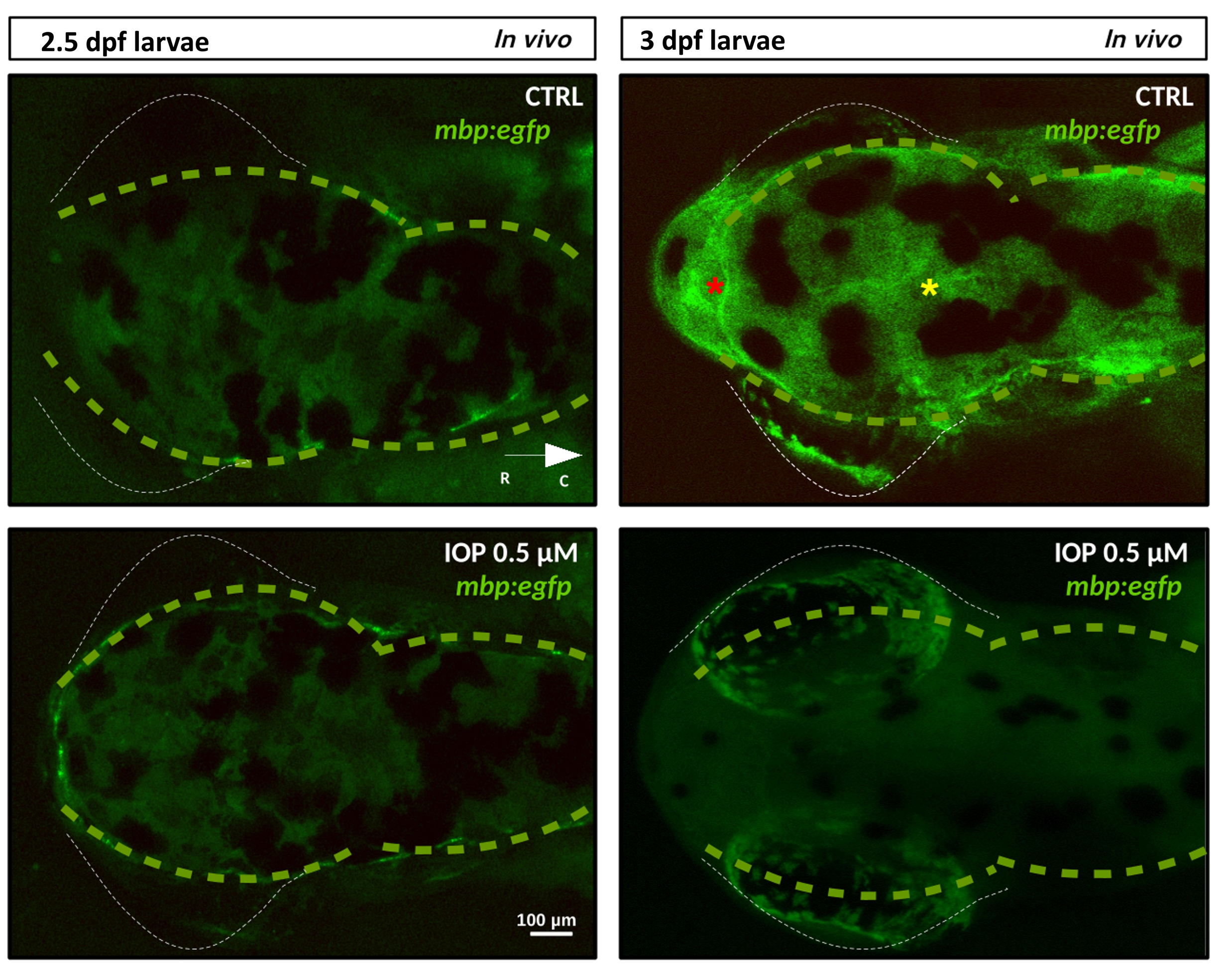

Supplement: S3 Fig — Control and IOP 0.5 μM-treated mbp:egfp transgenic zebrafish were photographed at 2.5 dpf and 3 dpf. At each time point, larvae were anesthetized with Tricaine mesylate (MS-222) 0.2 mg/ml for 45 seconds and fixed and orientated in agarose 1.5%. Confocal images were captured as previously indicated. After caption, animals were returned in a new chamber to their respective treatment. Given that capture was performed in live larvae, the orientation of the head was not always optimal. (TIF) [file pone.0256207.s003.tif]
