# Supplementary material for: Thyroid hormone deficiency during zebrafish development impairs central nervous system myelination
Source: PLoS One. 2021 Aug 17;16(8):e0256207. doi: 10.1371/journal.pone.0256207 (PMC8370640; doi:10.1371/journal.pone.0256207)
Supplement: S1 Table — (PDF) [file pone.0256207.s004.pdf]

Table 1. Real time PCR primers

Gene Target	Accession Number	Primer Sequence (5´- 3´)	Primer Position	Length (bp)	Ref
dio2	NM_212789.4	F: TCTGGAGGAGAGGATGTTTGC<br>R: CTCGTAGGACACACCGTAGG	exon 2	124	1
dio3a	NM_001256003.1	F: CGCTCGTGTGTCTGCTCATT<br>R: CAGAGACTCCCAGCTGAACA	exon 1	196	2
gh	NM_001020492.2	F: TGCAACACCTTCACCAGC<br>R: GCGTTTCATCTTTTCCCGTC	exon 2 - 3	150	*
tshb	NM_181494.2	F: CAGGGACAGTAACATAAAGGAG<br>R: CTGGGTAGGTGAAGTGAGG	exon 2 - 3	137	1
ttr	NM_001005598.2	F: CGCACACCTTTCCACCAG<br>R: TTGACGACCACAGCAGTTG	exon 3	122	1
sox10	NM_131875.1	F: TCAATATCCGCACCTGCAC<br>R: CGCTTATCCGTCTCGTTCAG	exon 2 - 3	82	*
mbpa	ENSDART00000052556.8	F: ATCTCGCTCTCCACCCAAAC<br>R: GCGACTGGTGAGTCTGTAGG	exon 2	142	3
mpz	NM_194361.2	F: ACCTGTGATGCCAAGAACC<br>R: TTGCCACAACGAGGATCA	exon 3 - 4	148	*
olig2	ENSDART00000060006.5	F: CGAGTGAACTGGAATAGCCTTAC<br>R: GCTCGTGTCAGAGTCCATG	exon 1	134	*
plp1b	NM_001005586.2	F: AGTAAAGCAGACGTTCGGG<br>R: ACACAATCAGCCAGATCAGAG	exon 3 - 4	107	*
lsm12b	NM_213148.1	F: AGTTGTCCCAAGCCTATGCAATCAG<br>R: CCACTCAGGAGGATAAAGACGAGTC	exon 3 - 4	300	2
actb1	NM_131031.2	F: TGAATCCCAAAGCCAACAGAG<br>R: CCAGAGTCCATCACAATACCAG	exon 3 - 4	139	*

* Designed for the present study.

1. Aoife E. Parsons, Anke Lange, Thomas H. Hutchinson, Shinichi Miyagawa, Taisen Iguchi, Tetsuhiro Kudoh, Charles R. Tyler, Expression dynamics of genes in the hypothalamic-pituitary-thyroid (HPT) cascade and their responses to 3,3′,5-triiodo-l-thyronine (T3) highlights potential vulnerability to thyroid-disrupting chemicals in zebrafish (*Danio rerio*) embryo-larvae, Aquatic Toxicology, Volume 225, 2020, 105547, ISSN 0166-445X, https://doi.org/10.1016/j.aquatox.2020.105547.

2. Lazcano I, Rodríguez-Ortiz R, Villalobos P, Martínez-Torres A, Solís-Saínz JC, Orozco A. Knock-Down of Specific Thyroid Hormone Receptor Isoforms Impairs Body Plan Development in Zebrafish. Front Endocrinol (Lausanne). 2019;10:156. Published 2019 Mar 14. doi:10.3389/fendo.2019.00156.

3. Walter KM, Dach K, Hayakawa K, Giersiefer S, Heuer H, et al. (2019) Ontogenetic expression of thyroid hormone signaling genes: An in vitro and in vivo species comparison. PLOS ONE 14(9): e0221230. https://doi.org/10.1371/journal.pone.0221230
