# Supplementary material for: Thyroid hormone deficiency during zebrafish development impairs central nervous system myelination
Source: PLoS One. 2021 Aug 17;16(8):e0256207. doi: 10.1371/journal.pone.0256207 (PMC8370640; doi:10.1371/journal.pone.0256207)
Supplement: Attachment — Submitted filename: Answers to Reviewers.docx [file pone.0256207.s006.docx]

**Answers to Review**

We thank the reviewers for their valuable comments and suggestions. In the new version of the manuscript, we have included additional experiments to address some of the concerns raised, as well as rewritten sections of the discussion. The changes are shown in red throughout the revised manuscript.

Reviewer #1:

1. Assessing oligodendrocyte development by qPCR is difficult to interpret because a number of the markers assessed are expressed in large populations of other cell types: olig2 in motor neurons, sox10 in all neural crest, mbpa in Schwann cells. The authors should take advantage of preexisting transgenic lines or published in situ constructs for these markers, which would be more suitable to address a developmental delay in oligodendrocytes specifically.

and

4. Assessing oligodendrocyte myelination in single oligodendrocytes through injection of mbp:egfp for mosaic labeling would be useful to determine whether there is a developmental delay (delayed transition to making myelin sheaths), or if fewer oligodendrocytes are being made in general.

We agree with the reviewer that qPCR is not the optimal way to follow up on oligodendrocyte development, considering also that the concentration of the target gene is diluted when mRNA is extracted from the whole embryo. In order to gain some insights regarding a possible delay in oligodendrocyte development due to T3 deficiency, we have included two additional experiments: 1) a follow-up on myelin formation using live *mbp:egfp* larvae at the onset of myelination and once myelin formation is ongoing; 2) immunostaining using an OPC marker to analyze if OPCs showed a developmental delay with T3 deficiency or if fewer OPCs transitioned to myelinating OLs. (lines 318-330 and 398-406).

2. While the difference in Black-Gold II stain in Figure 2 is obvious, it is impossible to see the difference in the spinal cord (Figure 3) due to the extensive background staining. The authors should perform cross sections of the stained tissue and quantify the signal specifically within the spinal cord.

We thank the reviewer for the comment. We have repeated the experiment in both, whole-mount preparations and cross-sections. The Black-Gold II staining was improved, resulting in images where myelin is more clearly observed; however, when we performed the Black-Gold II staining in cross-section, images were not optimal to accurately measure myelin content because sectioning harms the integrity of the tissue. Since the new whole-mount images depict myelin content quite unequivocally, we decided to just include these in the new version of the manuscript. Also, we have eliminated the Figure 3 from the original version of the manuscript and have included spinal cord images in the new Figures 2 and 3.

3. The Black-Gold stain in Figure 4 has so much background that I do not think it actually reflects myelination. At 3 dpf, there is very little myelin present in the brain, so I do not feel confident that the authors are actually quantifying myelin. The authors should take advantage of published in situ probes for mbpa, or rely solely on the mbp:egfp transgene.

The reviewer is right in that it is difficult to observe myelin in the cross-sections included in Figure 3 of the original version of the manuscript although the magnitude of magnification used while performing myelin determinations allowed the measurements reported in the Figure. However, given the comment and considering that no changes were observed in the analyzed parameters (perimeter and diameter), we decided to eliminate these data from the new version of the manuscript.

5. Given the morphological defects in the IOP-treated animals, the authors should use acetylated tubulin stain to assess whether axon number and/or organization is impaired in these animals.

We repeated all experiments and found that the IOP-treated animals for 7 days did not consistently show morphological defects. However, to attend the reviewer’s concern, we searched for an antibody to label acetylated tubulin. We finally decided against these experiments since tubulin is a very dynamic structure and we could not find a label that would depict an impairment on axonal organization or number.

MINOR:
Line 67: Myelin is not present in all vertebrates. The sentence should be clarified to state that myelination is the last major event to occur in all vertebrates for which axons are myelinated.

Corrected.

Lines 77-79: Mbp is also robustly expressed in Schwann cells. The major difference between central and peripheral myelin is the presence of Plp in oligodendrocytes and Mpz in Schwann cells.

According to several authors, mpb, mpz and plp1 are distributed in both the central and the peripheral nervous systems. We have corrected the text and included new references to support this notion (Brösamle C, Halpern ME. Characterization of myelination in the developing zebrafish. Glia. 2002 Jul;39(1):47-57. doi: 10.1002/glia.10088. PMID: 12112375).

Lines 338-339: Olig2 is not specific to oligodendrocyte precursors. It is also a primary marker of motor neurons (PMID: 12167410), which complicates the interpretation of the qPCR results as noted above.

Corrected.

Statistics: Why were medians compared rather than means?

While the graph type used (Box and Whiskers) for Figures 1, 4-6 and Supplementary Figure 2 depict medians in the middle of the box, the ANOVA analysis coupled with Tukey test always compares the means. We just used the Box and Whiskers graph type to better show the dispersion of the data. Although we could change the graph type if the reviewer insists, the statistical analysis results remain the same.

Reviewer #2:

1) Rationale for the parts of the nervous system is not clear.

We selected regions that included the forebrain, midbrain and hindbrain to have a more integrative picture of CNS myelination. We have now specified the location within the nervous system of the anatomical structures that were analyzed in this study (lines 32, 173-174).

2) Even if IOP is known to affect deiodinase activity and thyroid hormone synthesis , an embryonic treatment with IOP in zebrafish is not well linked with hypothyroidism. As hypothyroidism is not fully demonstrated, some conclusions are not supported by the data presented. Would it be possible to better show the hypothyroidism? Can T3 and T4 measurements be performed on embryos (whole or tissue) before and after IOP treatment to support the fundamental hypothesis of hypothyroidism? If not, can a T4 antibody be used? Or study of the thyroid gland? pituitary (TSH) or thyroid growth related genes?

In the present study, we aimed to reduce T3 bioavailability, rather than to induce a classical hypothyroid condition. While embryonic T4 is from maternal origin, most of the embryonic T3 is derived from T4 to T3 conversion. Impairing this conversion with IOP is the only available method to reduce T3 signaling at very early stages, before the embryonic thyroid gland is active. While measuring T4 would not offer proof of a T3 deficiency, measuring T3 would; however, very sensitive methods would be needed to indeed show this partial T3 reduction. To attend the reviewer’s concerns, we instead: 1) set up a protocol to stain and analyze larval cartilage, which is known to reflect the thyroidal status in mammals and fish. Indeed, we observed malformations in Meckel´s cartilage, revealed by Alcian blue staining (Figure 1A); 2) we measured additional TH-linked genes like *thsb*, *ttr* and *gh*. For these and all the genes selected to measure the thyroidal status, we designed new oligos improving their qPCR amplification efficiency. All experiments were repeated, increasing the total number of individuals analyzed per experiment. With these new conditions, we observed a clear TH regulatory pattern for both, *dio2*, *dio3* and *tshb* (Figure 1B), supporting the lower T3 availability in the larvae. We were unable to detect changes in *ttr* and gh, suggesting that these genes are not as sensitive to T3 fluctuations, further supporting that with IOP treatment we induced a rather mild T3 deficiency.

3) The article is mostly descriptive and lacks a functional part to make the data fully usable and understandable.

To address this concern, we included two experiments to gain some insights regarding a possible delay in oligodendrocyte development due to T3 deficiency: 1) a follow-up on myelin formation using live *mbp:egfp* larvae at the onset of myelination and once myelin formation is ongoing; 2) immunostaining using an OPC marker to analyze if OPCs showed a developmental delay with T3 deficiency or if fewer OPCs transitioned to myelinating OLs (lines 318-330 and 398-406).

4) Statistics: with n=3 for the gene exppressions for exemple. A comparison of more than two groups by mann whitney is not recommanded (ANOVA should be used but I don’t know if n=3 is enough for statistical power).

We thank the reviewer for noting the errors. As mentioned previously, we have repeated all statistical analyses with the suitable statistical tests, running ANOVAs in the experiments where more than 2 groups were compared. Also, the n value in each experiment was increased for a more robust analysis.

Genes are quantified and related to a total amount of RNA. However, the actin B1 gene is in the list of primers used. It is not clear whether this housekeeping gene has been used for relative quantification or not.

We have extended the section on the qPCR methodology for clarity in the new version of the manuscript (lines 137-145). We explain how absolute, not relative gene quantification is performed and the use of two reference genes as correction factors.

The authors assume that the decrease in dio3 is a sign of hypothyroidism but the increased expression at 3dpf of thrb, mpa at 7 days and the decrease in thraa and thrab do not support this hypothesis. It is also debatable whether these measurements are made on whole organisms. Only an overall net effect is therefore observed and correlations with tissue or even cellular events in the nervous system are hazardous, given that opposite cellular regulations can be observed, particularly during development. A discussion on tissue and cellular effects of thyroid hormone would be therefore interesting

As mentioned in the response of point 2, we have included other criteria to determine reduced T3 availability signs in the new version of the manuscript. We have also included a sentence (lines 361-364) to include the arguments raised. However, we decided against discussing on tissue and cellular effects of thyroid hormones in the developing nervous system of zebrafish, given that we do not have detailed results in the present study and that available published information is scarce.

It is quite surprising to see that the T3 treatment alone has not been carried out on gene quantification and it is not described whether the 50/60 embryos x3 come from 3 independent experiments or if they are from the same experiment.

In the newly performed experiments, we have included results from gene quantification of T3- (Figure 1) and IOP + T3-treated groups (Figures 1 and 4). We have also clarified that each pool represents an independent experiment (lines 582 and 615).

There is no functional experience on the consequences of a delay in myelination (movement for example) and rescue that the T3 treatment could bring. Furthermore, cross sections show a lack of effect of IOP treatment on myelinated axons. How then can this apparent contradiction be explained with the drop in myelin density observed with Black gold II marking? What are the effects of T3 and IOP+T3 treatment on these parameters?

Indeed, cross sections did not show a decrease in axonal diameter. In order to confirm these results, we repeated the experiment in both, whole-mount preparations and cross-sections. The Black-Gold II staining was improved, resulting in images where myelin is more clearly observed; however, when we performed the Black-Gold II staining in cross-section, images were not optimal to accurately measure myelin content because sectioning harms the integrity of the tissue. Since the new whole-mount images depict myelin content quite unequivocally, we decided to just include these in the new version of the manuscript. For this reason, we have eliminated the Figure 3 of the original version of the manuscript and have included new images in Figures 2 and 3. In them, we observe a clear decrement in myelin content after IOP treatment at 3 and 7 dpf. However, this decrement is not reflected in movement. In our hands, we found no differences between control and IOP-treated 7dpf zebrafish larvae in natural swimming behavior using single visual inspection and through a free software analysis (Kinovea, ToxTrac). The analysis of the effects of T3 and IOP+T3 was not possible because 7 dpf larvae died during the experimental treatment, as mentioned in the new version of the manuscript. We have included this information in lines 353-356.

Fig 6/ Video Suppl 4: the term positive control is not appropriate (solvant control). In the video the size is not the same than the others 3D reconstructions.

A scale bar should be present in all sections/pictures/video

We have corrected the video.
