# Supplementary material for: Thyroid hormone deficiency during zebrafish development impairs central nervous system myelination
Source: PLoS One. 2021 Aug 17;16(8):e0256207. doi: 10.1371/journal.pone.0256207 (PMC8370640; doi:10.1371/journal.pone.0256207)
Supplement: Attachment — Submitted filename: R2. Answers to Reviewers.docx [file pone.0256207.s007.docx]

Reviewer #1:

1. For the new Alcian blue stains (Figure 1A-B), the authors should show labeling and quantification of the cartilage malformation. To the general reader, this difference is not immediately apparent.

Figure 1 now includes an additional graph showing cartilage quantification of control and IOP-treated larvae. We have also included a sentence in the text to assist the reader (lines 588-589).

2. For all graphs, individual data points she be plotted within the bar graph or box/whisker plot to increase transparency of reporting.

In this R2 version of the manuscript, individual data points were plotted in all the graphs. The corresponding Figure Legends were modified accordingly.

3. The control images for CeP and D+DT+TeO in Figure 2 in the resubmission are not nearly as clear as Figure 2 in the original submission. Please provide representative images with less background stain.

We have taken new larvae photographs from the different treatments and were able to capture better representative images. We have included the new photographs in Figures 2 and 3.

MINOR:

1. Line 68: Myelination in mammals starts in the late stages of embryonic development, not during early stages of development.

We have corrected this error in the text (line 69).

2. Line 97-100. Need a citation or else need to state that this is the hypothesis.

We have rephrased this sentence, stating it as a hypothesis (lines 99-102).

3. Line 101 typo. Should read “post-fertilization in teleost”

4. Line 104 typo. Should read “later of larval”

We have corrected both typos.

5. Lines 126-127 are redundant with lines 121-122.

We understand that there may be a redundancy stating the guidelines for the care and use of laboratory animals; however, the journal specifies these requirements.

6. Lines 284/285, it would be helpful to have these structures defined for the first time here rather than in the methods.

The description of the studied structures was described in the Results section under “Quantification of myelin with Black-Gold II staining”, as suggested (lines 284-286).

7. Figure 5: need to label the transgene used and the developmental stage.

We have corrected the Figure Legend for Figure 5, adding the missing data.

Reviewer #2:

1. The results at 3 days are not always very convincing since only developmental delay could explain the absence of myelin in the fin.

We measured control and IOP-treated larvae at 3 and 7 dpf. We observed that at 3 dpf, the lengths are similar between both groups, supporting the notion that at that stage there is no overall delay in development and the absence of myelin is more specifically related to lower T3 bioavailability. However, 7 dpf IOP-treated larvae were significantly smaller than controls, as well as showed lower myelination levels. Altogether, our results show that although a developmental delay cannot be ruled out, lower T3 bioavailability does impair correct myelination. A paragraph has been added to the discussion of the R2 version of the manuscript (lines 347-354).

2. I personally find it very difficult to see a difference between BGII stained animals, without the fibers drawn in red in figures 2 and 3.

We have photographed again larvae from the different treatments and were able to capture better representative images. We have included the new photographs in Figures 2, 3 and 5.

3. IHC with NG2 on larvae in dorsal view seem diffuse and the question of specificity arises.

Figure 6 now includes a representative image of a negative control to show that the observed NG2 signal is indeed specific.

4. The authors also added experiments using the mbp:eGFP transgenic line. It would have been useful to confirm the data in Figure 3 with this model (given the paper by Jung et al 2010 and the specificity observed in the fin but not necessarily in the brain). Moreover, the signal seems very diffuse.

Jung et al (2010) reported EGFP^+^ labeling in *mbp:egfp* transgenic zebrafish in both, hindbrain and spinal cord starting at 5 dpf. We are here adding to those observations by describing EGFP+ signal in anterior brain regions which were not explored in the original manuscript. We decided to analyze myelination at 3 dpf because it represents the onset of this process. Since similar results were observed when analyzing IOP effects upon myelin content in 3 dpf larvae stained with Black-Gold II or injected with the *mbp:egfp* transgene, we did not consider that confirming the Black-Gold II data using the transgenic model at 7 dpf would add more information. To address the reviewer’s concern regarding the diffuse signal in Figure 3, we have included new representative images that have a better definition.

5. Finally the structures, especially the telencephalon are clearly different when comparing IOP, CTRL and T3 and here again a growth delay explaining the absence of myelin cannot be ruled out.

As mentioned in the response to point 1 of this rebuttal, lengths from control and IOP-treated larvae are similar at 3 dpf, arguing against the idea that the absence or decrease in myelin content is due to growth delay at this developmental stage. It is true however that the fluorescent signal in the telencephalon of control, IOP- and T3-treated larvae is different. As shown in Figure 5, a strong rhomboid-like structure is observed in control and T3-treated 3 dpf larvae, but it is not visible in those treated with IOP or IOP+T3. We have photographed Black-Gold II stained larvae in the same position as that used for *mbp:egfp* transgenic larvae and have added the images to Figure 5. It is clear with both myelin visualizing methods that the rhomboid-like structure is compromised in IOP-treated larvae, adding further support that lower T3 indeed reduces myelination. At 3 dpf myelin content is less abundant in other brain areas, like the tectum opticum or the cerebellar plate, making the impact of lower T3 availability less obvious.

6. line 227: This is not true that IOP and T3 treatment always induce gene expression going to opposite ways. For example, for dio2, they go the same sense. Actually this statement is only valid for dio3 and tshb expression.

We have corrected this statement in the R2 version of the manuscript (line 276).

7. Also in fig 4 it seems quite weard that T3+IOP is not more fluorescent than CTRL given the very high fluorescent signal in TeO.

We have included a new representative photograph for the *mbp:egfp* IOP+T3 in Figure 5, which is neater and has less autofluorescence.
